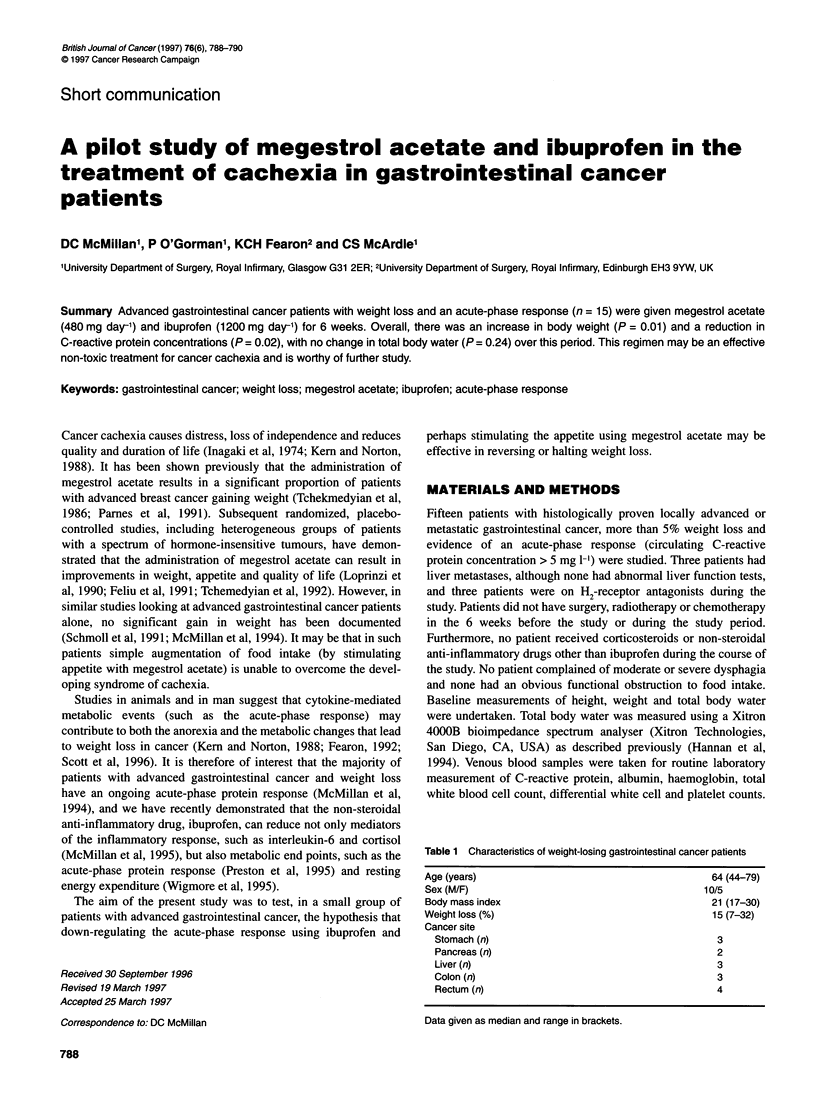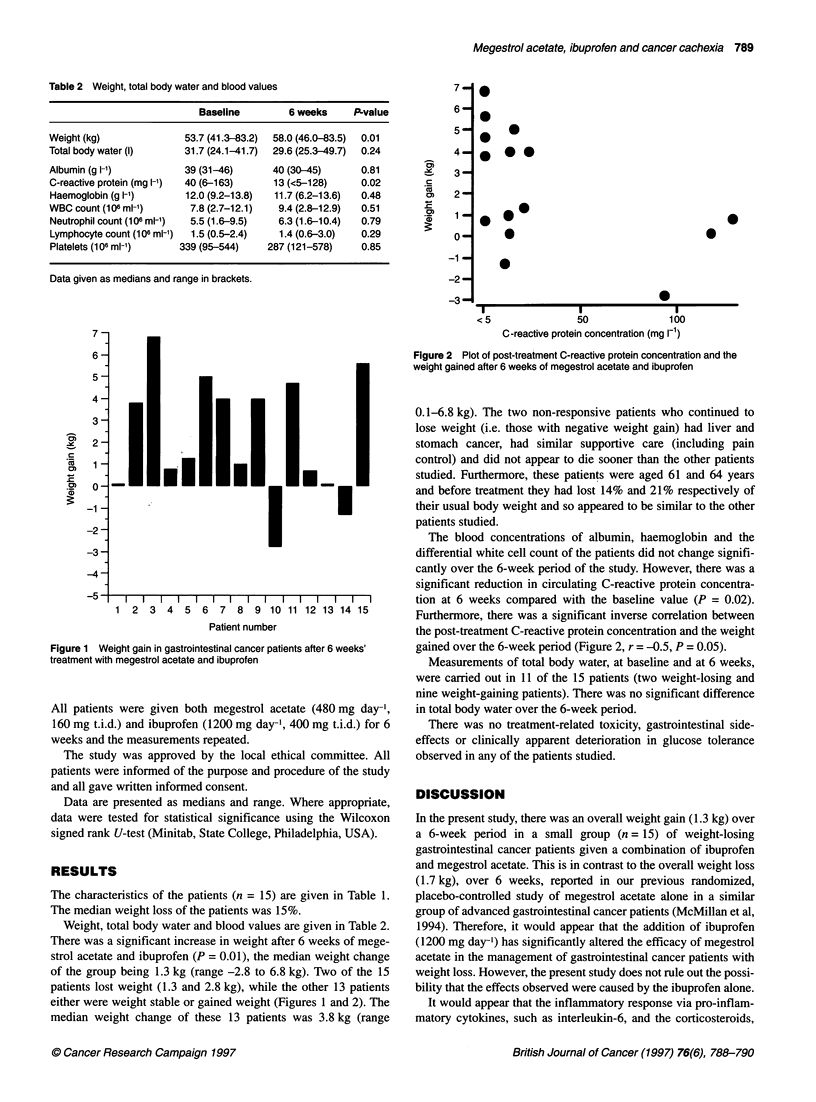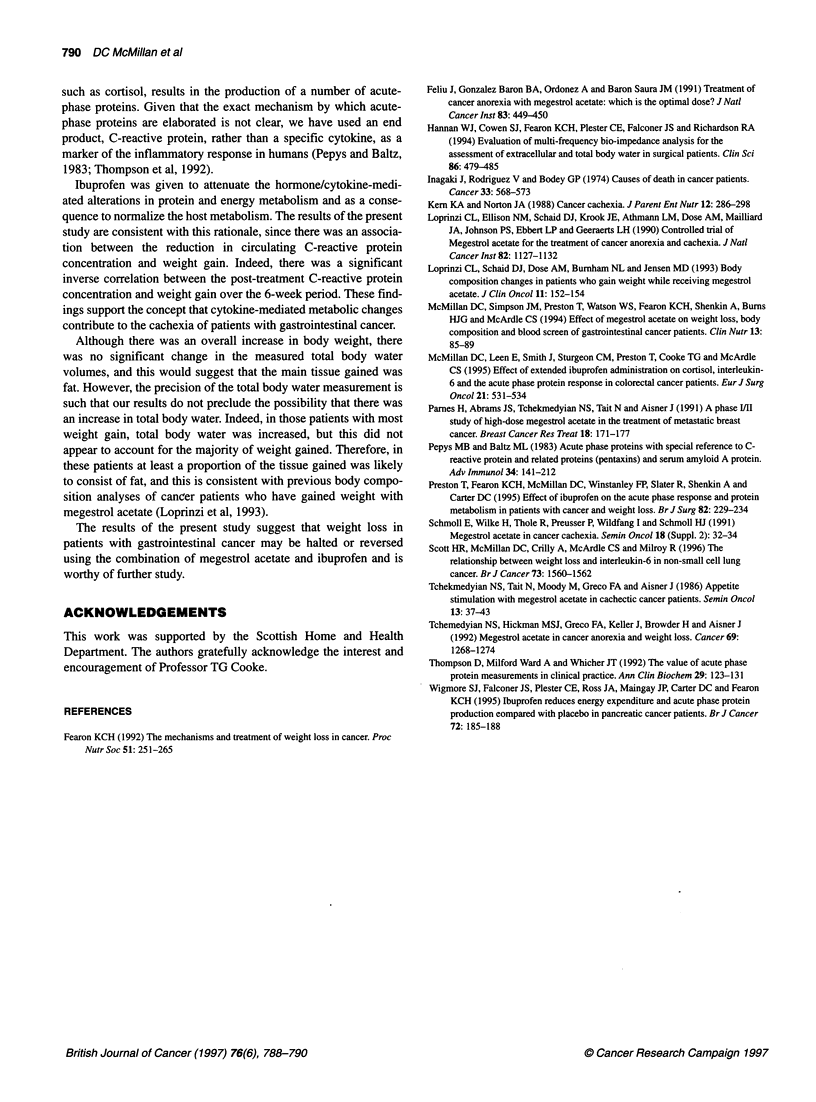# A pilot study of megestrol acetate and ibuprofen in the treatment of cachexia in gastrointestinal cancer patients.

**DOI:** 10.1038/bjc.1997.463

**Published:** 1997

**Authors:** D. C. McMillan, P. O'Gorman, K. C. Fearon, C. S. McArdle

**Affiliations:** University Department of Surgery, Royal Infirmary, Glasgow, UK.

## Abstract

Advanced gastrointestinal cancer patients with weight loss and an acute-phase response (n = 15) were given megestrol acetate (480 mg day(-1)) and ibuprofen (1200 mg day(-1)) for 6 weeks. Overall, there was an increase in body weight (P = 0.01) and a reduction in C-reactive protein concentrations (P = 0.02), with no change in total body water (P = 0.24) over this period. This regimen may be an effective non-toxic treatment for cancer cachexia and is worthy of further study.


British Journal of Cancer (1997) 76(6), 788–790
© 1997 Cancer Research Campaign

Short communication

### A pilot study of megestrol acetate and ibuprofen in the treatment of cachexia in gastrointestinal cancer patients

**DC McMillan[1], P O'Gorman[1], KCH Fearon[2] and CS McArdle[1]**

[1]University Department of Surgery, Royal Infirmary, Glasgow G31 2ER; [2]University Department of Surgery, Royal Infirmary, Edinburgh EH3 9YW, UK

**Summary** Advanced gastrointestinal cancer patients with weight loss and an acute-phase response ($n$ = 15) were given megestrol acetate (480 mg day$^{-1}$) and ibuprofen (1200 mg day$^{-1}$) for 6 weeks. Overall, there was an increase in body weight ($P$ = 0.01) and a reduction in C-reactive protein concentrations ($P$ = 0.02), with no change in total body water ($P$ = 0.24) over this period. This regimen may be an effective non-toxic treatment for cancer cachexia and is worthy of further study.

**Keywords:** gastrointestinal cancer; weight loss; megestrol acetate; ibuprofen; acute-phase response

Cancer cachexia causes distress, loss of independence and reduces quality and duration of life (Inagaki et al, 1974; Kern and Norton, 1988). It has been shown previously that the administration of megestrol acetate results in a significant proportion of patients with advanced breast cancer gaining weight (Tchekmedyian et al, 1986; Parnes et al, 1991). Subsequent randomized, placebo-controlled studies, including heterogeneous groups of patients with a spectrum of hormone-insensitive tumours, have demonstrated that the administration of megestrol acetate can result in improvements in weight, appetite and quality of life (Loprinzi et al, 1990; Feliu et al, 1991; Tchemedyian et al, 1992). However, in similar studies looking at advanced gastrointestinal cancer patients alone, no significant gain in weight has been documented (Schmoll et al, 1991; McMillan et al, 1994). It may be that in such patients simple augmentation of food intake (by stimulating appetite with megestrol acetate) is unable to overcome the developing syndrome of cachexia.

Studies in animals and in man suggest that cytokine-mediated metabolic events (such as the acute-phase response) may contribute to both the anorexia and the metabolic changes that lead to weight loss in cancer (Kern and Norton, 1988; Fearon, 1992; Scott et al, 1996). It is therefore of interest that the majority of patients with advanced gastrointestinal cancer and weight loss have an ongoing acute-phase protein response (McMillan et al, 1994), and we have recently demonstrated that the non-steroidal anti-inflammatory drug, ibuprofen, can reduce not only mediators of the inflammatory response, such as interleukin-6 and cortisol (McMillan et al, 1995), but also metabolic end points, such as the acute-phase protein response (Preston et al, 1995) and resting energy expenditure (Wigmore et al, 1995).

The aim of the present study was to test, in a small group of patients with advanced gastrointestinal cancer, the hypothesis that down-regulating the acute-phase response using ibuprofen and perhaps stimulating the appetite using megestrol acetate may be effective in reversing or halting weight loss.

*Received 30 September 1996*
*Revised 19 March 1997*
*Accepted 25 March 1997*

*Correspondence to:* DC McMillan

### MATERIALS AND METHODS

Fifteen patients with histologically proven locally advanced or metastatic gastrointestinal cancer, more than 5% weight loss and evidence of an acute-phase response (circulating C-reactive protein concentration > 5 mg l$^{-1}$) were studied. Three patients had liver metastases, although none had abnormal liver function tests, and three patients were on $H_2$-receptor antagonists during the study. Patients did not have surgery, radiotherapy or chemotherapy in the 6 weeks before the study or during the study period. Furthermore, no patient received corticosteroids or non-steroidal anti-inflammatory drugs other than ibuprofen during the course of the study. No patient complained of moderate or severe dysphagia and none had an obvious functional obstruction to food intake. Baseline measurements of height, weight and total body water were undertaken. Total body water was measured using a Xitron 4000B bioimpedance spectrum analyser (Xitron Technologies, San Diego, CA, USA) as described previously (Hannan et al, 1994). Venous blood samples were taken for routine laboratory measurement of C-reactive protein, albumin, haemoglobin, total white blood cell count, differential white cell and platelet counts.

**Table 1** Characteristics of weight-losing gastrointestinal cancer patients

| | |
|---|---|
| Age (years) | 64 (44–79) |
| Sex (M/F) | 10/5 |
| Body mass index | 21 (17–30) |
| Weight loss (%) | 15 (7–32) |
| Cancer site | |
| Stomach ($n$) | 3 |
| Pancreas ($n$) | 2 |
| Liver ($n$) | 3 |
| Colon ($n$) | 3 |
| Rectum ($n$) | 4 |

Data given as median and range in brackets.

**Table 2** Weight, total body water and blood values

| | Baseline | 6 weeks | *P*-value |
|---|---|---|---|
| Weight (kg) | 53.7 (41.3–83.2) | 58.0 (46.0–83.5) | 0.01 |
| Total body water (l) | 31.7 (24.1–41.7) | 29.6 (25.3–49.7) | 0.24 |
| Albumin (g $l^{-1}$) | 39 (31–46) | 40 (30–45) | 0.81 |
| C-reactive protein (mg $l^{-1}$) | 40 (6–163) | 13 (<5–128) | 0.02 |
| Haemoglobin (g $l^{-1}$) | 12.0 (9.2–13.8) | 11.7 (6.2–13.6) | 0.48 |
| WBC count ($10^6$ $ml^{-1}$) | 7.8 (2.7–12.1) | 9.4 (2.8–12.9) | 0.51 |
| Neutrophil count ($10^6$ $ml^{-1}$) | 5.5 (1.6–9.5) | 6.3 (1.6–10.4) | 0.79 |
| Lymphocyte count ($10^6$ $ml^{-1}$) | 1.5 (0.5–2.4) | 1.4 (0.6–3.0) | 0.29 |
| Platelets ($10^6$ $ml^{-1}$) | 339 (95–544) | 287 (121–578) | 0.85 |

Data given as medians and range in brackets.

**Figure 1** Weight gain in gastrointestinal cancer patients after 6 weeks' treatment with megestrol acetate and ibuprofen

All patients were given both megestrol acetate (480 mg $day^{-1}$, 160 mg t.i.d.) and ibuprofen (1200 mg $day^{-1}$, 400 mg t.i.d.) for 6 weeks and the measurements repeated.

The study was approved by the local ethical committee. All patients were informed of the purpose and procedure of the study and all gave written informed consent.

Data are presented as medians and range. Where appropriate, data were tested for statistical significance using the Wilcoxon signed rank *U*-test (Minitab, State College, Philadelphia, USA).

### RESULTS

The characteristics of the patients ($n$ = 15) are given in Table 1. The median weight loss of the patients was 15%.

Weight, total body water and blood values are given in Table 2. There was a significant increase in weight after 6 weeks of megestrol acetate and ibuprofen ($P$ = 0.01), the median weight change of the group being 1.3 kg (range −2.8 to 6.8 kg). Two of the 15 patients lost weight (1.3 and 2.8 kg), while the other 13 patients either were weight stable or gained weight (Figures 1 and 2). The median weight change of these 13 patients was 3.8 kg (range 0.1–6.8 kg). The two non-responsive patients who continued to lose weight (i.e. those with negative weight gain) had liver and stomach cancer, had similar supportive care (including pain control) and did not appear to die sooner than the other patients studied. Furthermore, these patients were aged 61 and 64 years and before treatment they had lost 14% and 21% respectively of their usual body weight and so appeared to be similar to the other patients studied.

**Figure 2** Plot of post-treatment C-reactive protein concentration and the weight gained after 6 weeks of megestrol acetate and ibuprofen

The blood concentrations of albumin, haemoglobin and the differential white cell count of the patients did not change significantly over the 6-week period of the study. However, there was a significant reduction in circulating C-reactive protein concentration at 6 weeks compared with the baseline value ($P$ = 0.02). Furthermore, there was a significant inverse correlation between the post-treatment C-reactive protein concentration and the weight gained over the 6-week period (Figure 2, $r = -0.5$, $P = 0.05$).

Measurements of total body water, at baseline and at 6 weeks, were carried out in 11 of the 15 patients (two weight-losing and nine weight-gaining patients). There was no significant difference in total body water over the 6-week period.

There was no treatment-related toxicity, gastrointestinal side-effects or clinically apparent deterioration in glucose tolerance observed in any of the patients studied.

### DISCUSSION

In the present study, there was an overall weight gain (1.3 kg) over a 6-week period in a small group ($n$ = 15) of weight-losing gastrointestinal cancer patients given a combination of ibuprofen and megestrol acetate. This is in contrast to the overall weight loss (1.7 kg), over 6 weeks, reported in our previous randomized, placebo-controlled study of megestrol acetate alone in a similar group of advanced gastrointestinal cancer patients (McMillan et al, 1994). Therefore, it would appear that the addition of ibuprofen (1200 mg $day^{-1}$) has significantly altered the efficacy of megestrol acetate in the management of gastrointestinal cancer patients with weight loss. However, the present study does not rule out the possibility that the effects observed were caused by the ibuprofen alone.

It would appear that the inflammatory response via pro-inflammatory cytokines, such as interleukin-6, and the corticosteroids,

© Cancer Research Campaign 1997

such as cortisol, results in the production of a number of acute-phase proteins. Given that the exact mechanism by which acute-phase proteins are elaborated is not clear, we have used an end product, C-reactive protein, rather than a specific cytokine, as a marker of the inflammatory response in humans (Pepys and Baltz, 1983; Thompson et al, 1992).

Ibuprofen was given to attenuate the hormone/cytokine-mediated alterations in protein and energy metabolism and as a consequence to normalize the host metabolism. The results of the present study are consistent with this rationale, since there was an association between the reduction in circulating C-reactive protein concentration and weight gain. Indeed, there was a significant inverse correlation between the post-treatment C-reactive protein concentration and weight gain over the 6-week period. These findings support the concept that cytokine-mediated metabolic changes contribute to the cachexia of patients with gastrointestinal cancer.

Although there was an overall increase in body weight, there was no significant change in the measured total body water volumes, and this would suggest that the main tissue gained was fat. However, the precision of the total body water measurement is such that our results do not preclude the possibility that there was an increase in total body water. Indeed, in those patients with most weight gain, total body water was increased, but this did not appear to account for the majority of weight gained. Therefore, in these patients at least a proportion of the tissue gained was likely to consist of fat, and this is consistent with previous body composition analyses of cancer patients who have gained weight with megestrol acetate (Loprinzi et al, 1993).

The results of the present study suggest that weight loss in patients with gastrointestinal cancer may be halted or reversed using the combination of megestrol acetate and ibuprofen and is worthy of further study.

### ACKNOWLEDGEMENTS

This work was supported by the Scottish Home and Health Department. The authors gratefully acknowledge the interest and encouragement of Professor TG Cooke.

### REFERENCES

Fearon KCH (1992) The mechanisms and treatment of weight loss in cancer. *Proc Nutr Soc* **51**: 251–265

Feliu J, Gonzalez Baron BA, Ordonez A and Baron Saura JM (1991) Treatment of cancer anorexia with megestrol acetate: which is the optimal dose? *J Natl Cancer Inst* **83**: 449–450

Hannan WJ, Cowen SJ, Fearon KCH, Plester CE, Falconer JS and Richardson RA (1994) Evaluation of multi-frequency bio-impedance analysis for the assessment of extracellular and total body water in surgical patients. *Clin Sci* **86**: 479–485

Inagaki J, Rodriguez V and Bodey GP (1974) Causes of death in cancer patients. *Cancer* **33**: 568–573

Kern KA and Norton JA (1988) Cancer cachexia. *J Parent Ent Nutr* **12**: 286–298

Loprinzi CL, Ellison NM, Schaid DJ, Krook JE, Athmann LM, Dose AM, Mailliard JA, Johnson PS, Ebbert LP and Geeraerts LH (1990) Controlled trial of Megestrol acetate for the treatment of cancer anorexia and cachexia. *J Natl Cancer Inst* **82**: 1127–1132

Loprinzi CL, Schaid DJ, Dose AM, Burnham NL and Jensen MD (1993) Body composition changes in patients who gain weight while receiving megestrol acetate. *J Clin Oncol* **11**: 152–154

McMillan DC, Simpson JM, Preston T, Watson WS, Fearon KCH, Shenkin A, Burns HJG and McArdle CS (1994) Effect of megestrol acetate on weight loss, body composition and blood screen of gastrointestinal cancer patients. *Clin Nutr* **13**: 85–89

McMillan DC, Leen E, Smith J, Sturgeon CM, Preston T, Cooke TG and McArdle CS (1995) Effect of extended ibuprofen administration on cortisol, interleukin-6 and the acute phase protein response in colorectal cancer patients. *Eur J Surg Oncol* **21**: 531–534

Parnes H, Abrams JS, Tchekmedyian NS, Tait N and Aisner J (1991) A phase I/II study of high-dose megestrol acetate in the treatment of metastatic breast cancer. *Breast Cancer Res Treat* **18**: 171–177

Pepys MB and Baltz ML (1983) Acute phase proteins with special reference to C-reactive protein and related proteins (pentaxins) and serum amyloid A protein. *Adv Immunol* **34**: 141–212

Preston T, Fearon KCH, McMillan DC, Winstanley FP, Slater R, Shenkin A and Carter DC (1995) Effect of ibuprofen on the acute phase response and protein metabolism in patients with cancer and weight loss. *Br J Surg* **82**: 229–234

Schmoll E, Wilke H, Thole R, Preusser P, Wildfang I and Schmoll HJ (1991) Megestrol acetate in cancer cachexia. *Semin Oncol* **18** (Suppl. 2): 32–34

Scott HR, McMillan DC, Crilly A, McArdle CS and Milroy R (1996) The relationship between weight loss and interleukin-6 in non-small cell lung cancer. *Br J Cancer* **73**: 1560–1562

Tchekmedyian NS, Tait N, Moody M, Greco FA and Aisner J (1986) Appetite stimulation with megestrol acetate in cachectic cancer patients. *Semin Oncol* **13**: 37–43

Tchemedyian NS, Hickman MSJ, Greco FA, Keller J, Browder H and Aisner J (1992) Megestrol acetate in cancer anorexia and weight loss. *Cancer* **69**: 1268–1274

Thompson D, Milford Ward A and Whicher JT (1992) The value of acute phase protein measurements in clinical practice. *Ann Clin Biochem* **29**: 123–131

Wigmore SJ, Falconer JS, Plester CE, Ross JA, Maingay JP, Carter DC and Fearon KCH (1995) Ibuprofen reduces energy expenditure and acute phase protein production compared with placebo in pancreatic cancer patients. *Br J Cancer* **72**: 185–188

© Cancer Research Campaign 1997